# A Comparative Analysis between Ultrasound-Guided and Conventional Distal Transradial Access for Coronary Angiography and Intervention

**DOI:** 10.1155/2020/7342732

**Published:** 2020-09-08

**Authors:** Shinsuke Mori, Keisuke Hirano, Masahiro Yamawaki, Norihiro Kobayashi, Yasunari Sakamoto, Masakazu Tsutsumi, Yohsuke Honda, Kenji Makino, Shigemitsu Shirai, Yoshiaki Ito

**Affiliations:** Department of Cardiology, Saiseikai Yokohama City Eastern Hospital, Yokohama, Japan

## Abstract

**Objectives:**

To compare feasibility and safety between ultrasound-guided and conventional distal transradial access (dTRA).

**Background:**

Distal transradial access, a new technique for coronary angiography (CAG) and percutaneous coronary interventions (PCI), is safe and feasible and will become popular worldwide. Ultrasound-guided dTRA has been advocated to reduce failure rate and access-site complications. However, to date, the comparison of feasibility and safety between ultrasound-guided and conventional dTRA has not been reported.

**Method:**

Overall, 137 patients (144 procedures) who underwent CAG or PCI using dTRA between September 2018 and February 2019 were investigated. These patients were classified into two groups: C (dTRA with conventional punctures; 76 patients, 79 procedures) and U (dTRA with ultrasound-guided punctures; 61 patients, 65 procedures) groups. Successful procedural rate, procedural outcomes, and complication rate during hospital stays were compared between the two groups.

**Results:**

The procedural success rate was significantly higher in the U group than in the C group (97% vs. 87%, *P*=0.0384). However, the rate of PCI, puncture time, total fluoroscopy time, the volume of contrast medium, the rate of access-site ecchymosis, and incidence of nerve disorder were similar between the two groups. Additionally, radial artery occlusion after the procedure did not occur in this study.

**Conclusion:**

The ultrasound-guided dTRA for CAG or PCI was associated with a lower failure rate than conventional dTRA. However, there were no significant differences in puncture time and complication rate between the two procedures.

## 1. Introduction

The feasibility and safety of distal transradial access (dTRA) for coronary angiography (CAG) or percutaneous coronary intervention (PCI) has been reported recently, and it is expected that dTRA is going to be popular worldwide [1–3]. One of the advantages of dTRA is the reduction of puncture site complications such as bleeding and radial artery occlusion (RAO) [1]. However, the puncture of the distal radial artery is supposedly more difficult than the puncture of the radial artery, since the former is a tiny vessel [4]. Hadjivassiliou et al. advocated the use of ultrasound-guided dTRA, which is expected to reduce failure and complication rates [5]. However, to date, the comparison of feasibility and safety between ultrasound-guided and conventional dTRA has not been reported. Thus, we compared the successful procedural rate, procedural outcomes, and complication rate between ultrasound-guided and conventional dTRA.

## 2. Materials and Methods

### 2.1. Patients

**Figure 1:**
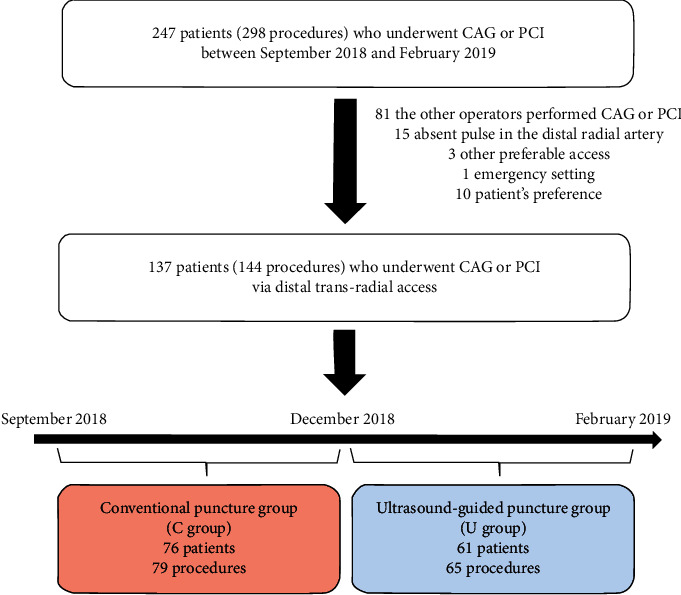
Study flowchart. CAG: coronary angiography; PCI: percutaneous coronary intervention.

This study recruited the patients who underwent CAG or PCI with dTRA between September 2018 and February 2019 at the Saiseikai Kanagawa Prefecture Hospital. Specifically, conventional dTRA was performed between September 2018 and November 2018, and ultrasound-guided dTRA was performed between December 2018 and February 2019. Of the 247 eligible patients (298 procedures), 166 consecutive patients (185 procedures) were assigned to the experienced interventional cardiologist (Dr. SM) operation program. In total, 137 patients (144 procedures) were considered suitable to undergo dTRA for CAG or PCI and further analyzed in this study. These patients were classified into two groups: C (dTRA with conventional punctures; 76 patients, 79 procedures) and U (dTRA with ultrasound-guided punctures; 61 patients, 65 procedures) groups. The remaining 29 patients did not undergo dTRA due to various reasons: the absence of a pulse in the distal radial artery (*n* = 15), other preferable access for complex PCI (*n* = 3), emergency setting (*n* = 1), and patient's preference (*n* = 10) (Figure 1). The evaluation criteria of the present study were the success rate of the procedure, puncture and procedural times, contrast volume, radiation dose and time, and complication rate during hospital stays. The protocol of the present study was conducted in accordance with the Declaration of Helsinki, and written informed consent was obtained from all patients who participate in this study.

### 2.2. Intervention

**Figure 2:**
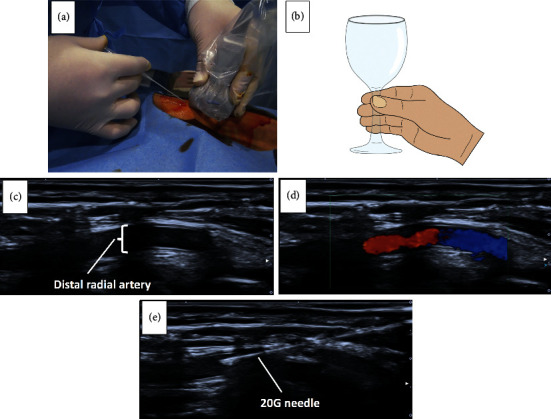
(a) A puncture with a 20 G needle under the long-axis ultrasound guidance. (b) The way of using the patient's hand that has a wineglass makes us puncture under ultrasound guidance easily. (c) Long-axis ultrasound shows the distal radial artery. (d) The Doppler-ultrasound shows the blood flow in the radial artery. (e) Ultrasound shows the puncture with a 20 G needle.

The region around the anatomical snuffbox was prepped and covered with a sterile material, and local anesthesia around the puncture site was administered with 5.0 mL of 1.0% lidocaine. The distal radial artery was punctured by the conventional method, and a 20-gauge (G) puncture needle was used in the same manner as that of a transradial access. The ultrasound-guided puncture was performed with a 20 G needle under the long-axis ultrasound guidance (Figures 2(a), 2(c)–2(e)). The nondominant hand of the operator held the ultrasound transducer, which was placed inside a sterile sheath, while the dominant hand held the 20 G needle. The needle punctured at an angle of 30–45° to the skin and was advanced under the ultrasound guidance until its entry into the distal radial artery was confirmed in the long axis view. The way of using the patient's hand that has a wineglass makes us puncture easily under ultrasound guidance (Figure 2(b)). After a successful puncture, a 0.021-inch plastic-type mini guidewire attached to the Terumo sheath (Terumo, Tokyo, Japan) was inserted. A 4, 5, or 6 Fr sheath was inserted over the guidewire. After the administration of isosorbide dinitrate and heparin (3000 units for CAG and 5000 units for PCI) intraarterially, CAG and PCI were performed as per protocol. For hemostasis, the hand around the puncture site was wrapped with a semipermeable polyurethane membrane (Tegaderm; 3M Healthcare, Germany) to protect the skin. The puncture site was compressed with a hemostasis device (STEPY; NICHIBAN, Tokyo, Japan), and the gauze was folded to form a cylinder and fixed to the top of the STEPY with a few turns of cohesive elastic bandage. The hemostasis continued for four hours. Ultrasound imaging unit (Viamo™, Canon Medical Systems, Tokyo, Japan) with an 8.0 MHz linear probe (PLT-1204BT transducer) was used in this study.

### 2.3. Definitions

Procedural failure implied switching to another puncture site. When a conventional dTRA was performed, any requirements to switch to ultrasound-guided dTRA were regarded as a procedural failure. The puncture was defined as the duration between draping with a sterile material to sheath insertion, while the procedural time was defined as the duration between the sheath insertion and sheath removal. Complications included access-site ecchymosis, major or minor hemorrhage, nerve disorder, and the occurrence of RAO. Minor and major bleeding implied Bleeding Academic Research Consortium (BARC) type 1 or 2 and type 3 or 5 bleeding, respectively [6].

### 2.4. Statistical Analysis

All statistical analyses were performed by the JMP software (version 13, SAS Institute, Cary, NC, USA). Categorical variables are expressed as frequencies, while continuous variables with normal distribution are expressed as mean ± standard deviation. Categorical variables were compared with the chi-square test or Fisher exact test, while continuous variables were compared with the unpaired Student's *t*-test or the Mann–Whitney *U* test. In all analyses, Poisson (*P*) value < 0.05 was considered statistically significant.

## 3. Results

### 3.1. Patients and Procedural Characteristics

In this study, 137 patients (144 procedures) were investigated. Patients and procedural characteristics are shown in Table 1. The mean age was significantly higher in the C group than in the U group (74.1 ± 9.6 years vs. 70.4 ± 10.5 years, *P*=0.03). There was no significant difference in the proportion of male gender, body mass index, the rate of diabetes mellitus, the rate of chronic kidney disease, the rate of dialysis, the use of left-handed access, sheath size, and the rate of PCI between the two groups.

### 3.2. Study Outcomes

**Figure 3:**
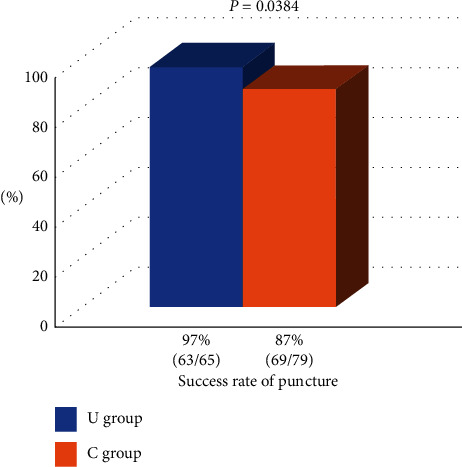
The success rate of puncture.

**Figure 4:**
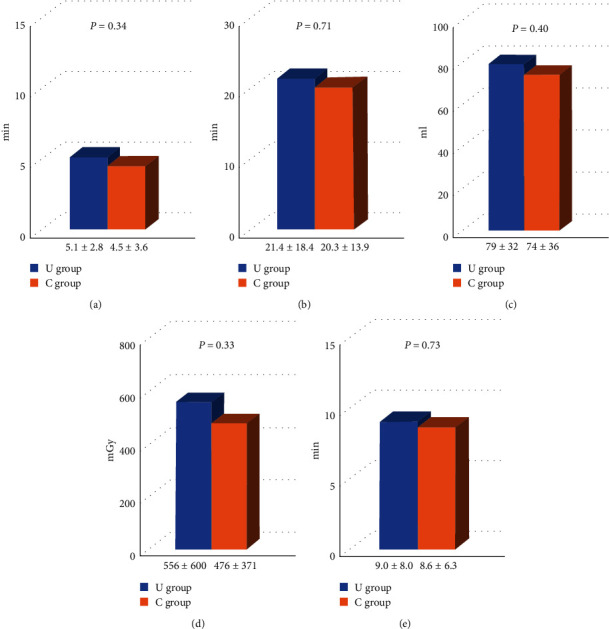
Procedural outcomes. (a) Puncture time. (b) Procedural time. (c) Contrast volume. (d) Radiation dose. (e) Radiation time.

**Figure 5:**
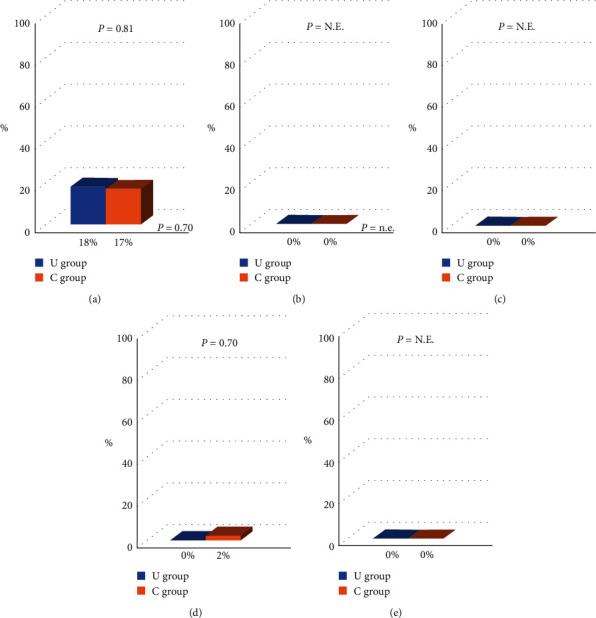
Complication rate. RAO: radial artery occlusion. (a) Access-site ecchymosis. (b) Minor bleeding. (c) Major bleeding. (d) Nerve disorder. (e) RAO.

The procedural success rate was significantly higher in the U group than in the C group (97% vs. 87%, *P*=0.0384) (Figure 3). The puncture and procedural times were similar between the U and C groups (5.1 ± 2.8 min vs. 4.5 ± 3.6 min, *P*=0.34 and 21.4 ± 18.4 min vs. 20.3 ± 13.9 min, *P*=0.71, respectively). The volume of contrast medium (79 ± 32 ml vs. 74 ± 36 ml, *P*=0.40), radiation dose (556 ± 600 mGy vs. 476 ± 371 mGy, *P*=0.33), and time (9.0 ± 8.0 min vs. 8.6 ± 6.3 min, *P*=0.73) were also similar between the two groups (Figure 4). Regarding complications, the access-site ecchymosis and neuropathy were similar between the two groups (18% vs. 17%, *P*=0.81, and 0% vs. 2%, *P*=0.70, respectively). Radial artery occlusion and minor or major bleeding after the procedure did not occur in this study (Figure 5).

**Figure 6:**
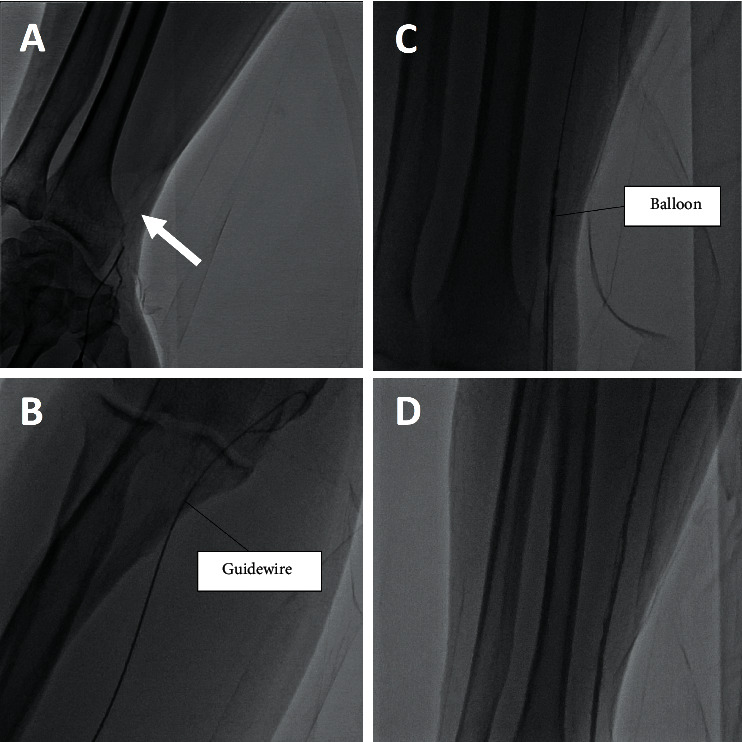
(a) Angiography from the distal radial artery shows the total occlusion of the radial artery (arrow). (b) Guidewire advance into the brachial artery. (c) After an exchange to 0.014-inch guidewire, balloon dilatation is performed. (d) Angiography from the distal radial artery shows the patency of the radial artery after the procedure.

In total, 7 cases were switched from conventional puncture to ultrasound-guided puncture, and procedures in all these cases were completed via ultrasound-guided dTRA. A representative case is described in the following. One patient had a chest pain due to a left anterior descending stenosis that was detected by coronary computed tomography angiography. The pulse of the bilateral distal radial artery was weak since the patient underwent CAG or PCI multiple times. Initially, a conventional puncture was attempted but it failed. Therefore, we switched to ultrasound-guided dTRA. The Doppler-ultrasound revealed the blood flow in the left distal radial artery, and thus, it was easy to puncture the distal radial artery under ultrasound guidance. Angiography after puncture revealed RAO (Figure 6(a)). The guidewire was passed through the RAO with a drilling technique (Figure 6(b)), and balloon dilatation was performed (Figure 6(c)). After the PCI procedure, angiography showed good patency of the radial artery (Figure 6(d)).

## 4. Discussion

This study is, to the best of our knowledge, the first report on the comparison between ultrasound-guided and conventional dTRA for CAG or PCI. This study demonstrated that ultrasound-guided dTRA for CAG or PCI can improve the rate of successful puncture, even though there were no significant differences in procedural outcomes and complication rates.

TRA for coronary diagnosis or revascularization procedure is progressively used worldwide since it is associated with lower bleeding and vascular complications than trans-femoral access [7]. However, specific complications of TRA exist; for instance, RAO can be observed in up to 13.7% of cases despite many precautionary measures [8, 9]. Regarding the occurrence of RAO, its future use as an access site for CAG or PCI, a conduit for coronary bypass grafting, or for fistula formation in hemodialysis patients is limited. Additionally, RAO is associated with a potential risk of hand ischemia [10, 11]. Kiemeneij first introduced the usefulness and safety of coronary catheterization via dTRA in 2017 [1], and yet, the occurrence of RAO after dTRA has never been reported to date [1, 4, 12]. Even if by chance RAO occurs, the flow to the thumb will still be maintained via the superficial palmar arch, thereby preventing hand ischemia and disability. However, the puncture of the distal radial artery is supposedly more difficult than the puncture of the radial artery, because the diameter of the distal radial artery in the anatomical snuffbox is significantly smaller than the diameter of the proximal radial artery [2, 4]. Generally, ultrasound-guided procedures such as puncture, injection, aspiration, and biopsy are frequently performed because the safety of the procedure is ensured by gaining positional information such as the location of the front-end of the device with ultrasound. Ultrasound guidance is particularly useful during arterial catheterization in patients with execrable situations such as obesity, abnormal anatomy, and hypotension with a weak pulse [13]. Therefore, for radial artery cannulation, there is category A, level 1 support for the use of ultrasound to improve first-pass success [14]. Moreover, it has been reported that ultrasound-guided TRA could be associated with a lower failure rate than conventional TRA (12% vs. 20%, *P*=0.012) [15]. This observation is in line with the findings of this study that showed ultrasound-guided dTRA was associated with a lower failure rate than conventional dTRA (3% vs. 13%, *P*=0.038). The failure rate of the conventional dTRA was 13% in this study, which is not much higher than the previously reported 11% failure rate [1]. It is worth mentioning that the failure rate could have been influenced by the selection criteria of this study, which did not exclude patients with weak distal radial artery pulse. On the other hand, there were no significant differences in the puncture time and complication rate between the conventional and ultrasound-guided dTRA. Such results were in line with the findings of a previous study that compared ultrasound-guided and conventional TRA [15].

Another potential advantage of ultrasound-guided dTRA is that it enabled us to puncture the distal radial artery even when the distal radial artery is not suitable for the conventional dTRA procedure. In the present study, switching to ultrasound-guided puncture from conventional puncture was required in 7 cases, and procedures in all these cases were completed via ultrasound-guided dTRA. The pulse of the distal radial artery can become weak after attempts of unsuccessful conventional puncture, because hematoma or vessel spasm can occur. In such situations, we could puncture the distal radial artery as far as the blood flow is visible with the Doppler-ultrasound.

As such, if an ultrasound machine is available in a catheterization laboratory, ultrasound-guided dTRA is preferred.

### 4.1. Limitations

This study has a few limitations. It is a single-center study and a single operator performed both conventional and ultrasound-guided dTRA. In addition, grouping according to the time period may have potentially led to bias. Moreover, the study was conducted on a small sample, and thus, further large-scale studies are needed to validate the findings of this study.

## 5. Conclusion

The ultrasound-guided dTRA for CAG or PCI was associated with a lower failure rate than conventional dTRA. However, there were no significant differences in puncture time and complication rate between the two procedures.

## Figures and Tables

**Table 1:** Patient and procedural characteristics.

	U group (61 patients, 65 procedures)	C group (76 patients, 79 procedures)	*P* value
Age, years	70.4 ± 10.5	74.1 ± 9.6	0.03
Male, %	42 (69)	53 (70)	0.91
Height, cm	161.4 ± 10.4	162.1 ± 9.0	0.67
Weight, kg	64.6 ± 16.1	62.0 ± 11.3	0.27
BMI, kg/m^2^	25.0 ± 4.0	23.8 ± 3.5	0.08
Smoking, %	14 (23)	19 (25)	0.86
Hypertension, %	48 (79)	60 (79)	0.97
Diabetes mellitus, %	30 (49)	29 (38)	0.20
Dyslipidemia, %	45 (74)	52 (68)	0.49
eGFR, ml/min/1.73 m^2^	62.8 ± 26.6	60.9 ± 21.6	0.65
CKD, %	22 (36)	29 (38)	0.80
Hemodialysis, %	6 (10)	4 (5)	0.31
PAD, %	5 (8)	4 (5)	0.49
CVD, %	4 (7)	4 (5)	0.75
Prior MI, %	6 (10)	6 (8)	0.69
Aspirin, %	53 (87)	62 (82)	0.40
Thienopyridine, %	42 (69)	55 (72)	0.65
Anticoagulant, %	5 (8)	7 (9)	0.83
Left hand, %	17 (27)	23 (29)	0.60
Sheath size			
4 Fr, %	0	2 (3)	0.32
5 Fr, %	57 (88)	64 (81)	
6 Fr, %	8 (12)	13 (16)	
CAG, %	45 (69)	55 (70)	0.96
PCI, %	20 (31)	24 (30)	

BMI, body mass index; CKD, chronic kidney disease; PAD, peripheral artery disease; CVD, cerebral vascular disease; MI, myocardial infarction; CAG, coronary angiography; PCI, percutaneous coronary intervention; eGFR, estimated glomerular filtration rate.

## Data Availability

No data were used to support this study.
